# Situs inversus and bariatric surgery: A challenge for the surgical team

**DOI:** 10.1016/j.amsu.2021.102972

**Published:** 2021-11-01

**Authors:** Juan F. Zavalza, Gabriel A. Molina, Omar A. Paipilla, Miriam Gil, Karla Rosales

**Affiliations:** Hospital Ciba, Tijuana, Mexico

**Keywords:** Situs inversus, Sleeve gastrectomy, Bariatric surgery

## Abstract

**Introduction and Importance:**

Obesity is a worldwide pandemic and is closely associated with an increased risk of comorbidities and overall mortality. Surgery has emerged as an essential strategy to ameliorate obesity-attributable comorbidities and as a powerful weight-loss tool. Due to the increasing number of obese patients predicted to elect surgery, individuals with rare anomalies such as situs inversus can be expected.

**Case presentation:**

We present the case of a 49-year-old woman with situs inversus with levocardia. She had a high BMI and was admitted for bariatric surgery. After several adjustments to our technique, the procedure was completed without complications.

**Clinical discussion:**

Laparoscopic bariatric surgeries are highly demanding; variations from the normal anatomy could challenge the medical team.

**Conclusion:**

Preoperative diagnosis and highly trained surgeons is of paramount importance to adequately treat every patient.

## Highlight

• Perioperative assessment of every patient is critical to ensure favorable results
• The experience and training of the surgical team will define the patient outcome
• Due to the increasing number of obese patients predicted to elect surgery, individuals with rare anomalies such as situs inversus can be expected

## 1 Introduction

Obesity is increasingly a major threat to human health in the developed and developing world. Bariatric surgery is currently considered the most efficacious treatment with the best long-term outcomes [1,2]. And since the prevalence of obesity has risen, many obese patients with rare genetic conditions such as Situs inversus have sought surgical consultation [1]. Situs inversus is a rare genetic condition characterized by the transposition of the viscera, despite the low prevalence of this condition and the problematic anatomic considerations [3,4]. These diseases must highlight the difficulty in performing laparoscopic operations on patients with mirror-image anatomy [3,5]. Therefore, preoperative planning and diagnosis are of paramount importance [6].

We present the case of a 49-year-old woman with a medical history of obesity and situs inversus with levocardia. After successful surgery, the patient completely recovered.

This manuscript has been reported in line with the SCARE 2020 criteria [12].

## 2 Case report

**Fig. 1:**
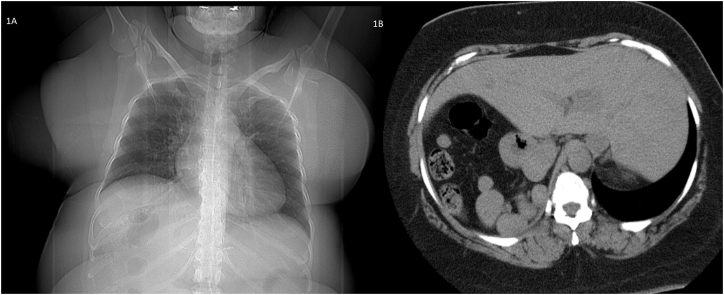
A: Chest X-ray. The heart is seen in normal position; B: Abdominal CT: Liver I noticed in the left side of the abdomen.

**Fig. 2:**
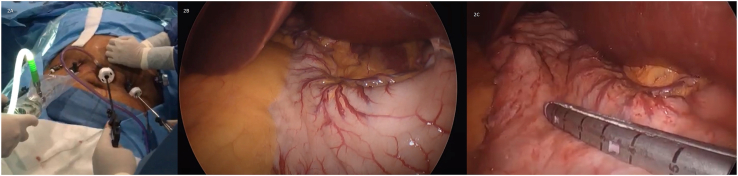
A: Trocar Placement on the patient with Situs Inversus; B: Greater curvature of the stomach on the left side of the patient; C: Gastric sleeve is being completed using a stapler.

Patient is a 49-year-old woman with a medical history of situs inversus with levocardia. She became aware of her condition ten years ago when she needed emergency surgery due to pain in her upper left abdomen; cholecystitis was detected, and she was successfully treated. During these last years, her weight has become a problem that has made it difficult to exercise or even walk. Therefore, she was admitted to our bariatric clinic. The patient had a BMI of 49.1 kg/m2, and fortunately, she didn't have any metabolic problems like diabetes, hypertension, or reflux. In addition, she did not suffer from other issues like bronchiectasis or sinusitis. Complementary exams (hematocrit and blood chemistry) were requested and came back normal; thus, she entered our bariatric program, and a gastric sleeve was planned. A preoperative CT scan of the abdomen confirmed the reversed position of the organs, and several adjustments were made to perform this unusual surgery (Fig. 1A and B). The surgical team handled the operation using a mirror image of the usual laparoscopic technique with the patient's reversed anatomy knowledge. The monitor was placed on the patient's right side, the surgeon stood on the left side, and the assistant on the right. We set a 10mm supraumbilical port for the staples and three 5 mm ports, one on the midclavicular line on the left side of the abdomen, one on the midclavicular line on the right side of the abdomen for the camera, and the last 5mm port on the right anterior axillary line for the traction of the omentum. As per routine, a 36-French Bougie was inserted under direct observation to achieve stomach decompression and then withdrawn upwards into the gastroesophageal junction. The incisura was identified, and a small window was opened on the lesser sac 5 cm away from the incision. Using a 5 mm ultrasound energy device, the omentum was detached from the greater curvature, and the dissection continued upwards, taking care of the short vessels and mobilizing the fundus. The bougie was advanced up to the first part of the duodenum, and under direct observation and using a surgical stapler (Waston Medical, Jiangsu Province, China), the sleeve was completed (Fig. 2A and B,2C and Fig. 3A). The staple line was then reinforced using a non-absorbable monofilament suture. A methylene blue test was performed, and no leaks were identified. Her postoperative course was uneventful; another leak test was performed on her second postoperative using fluoroscopy, which was negative, and she was discharged without any complications (Fig. 3B). On follow-up controls, the patient is doing well.

**Fig. 3A:**
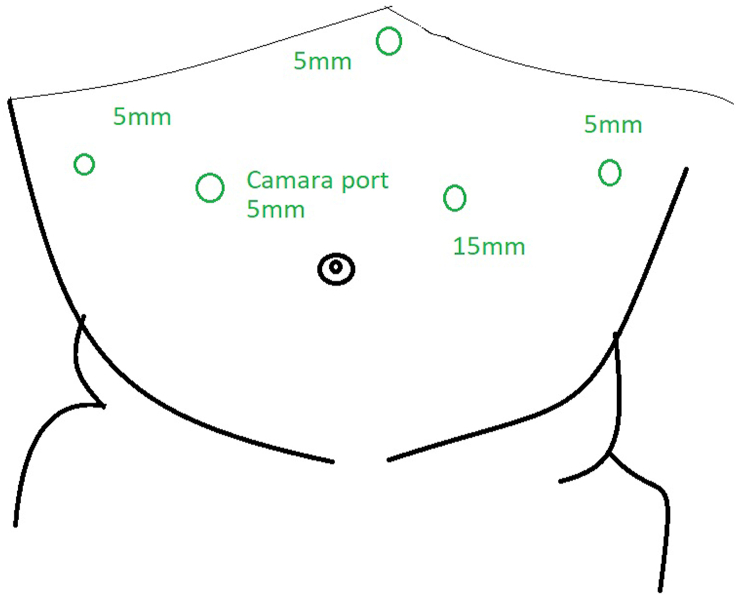
Diagram of port placement in a situs inversus patient.

**Fig. 3B:**
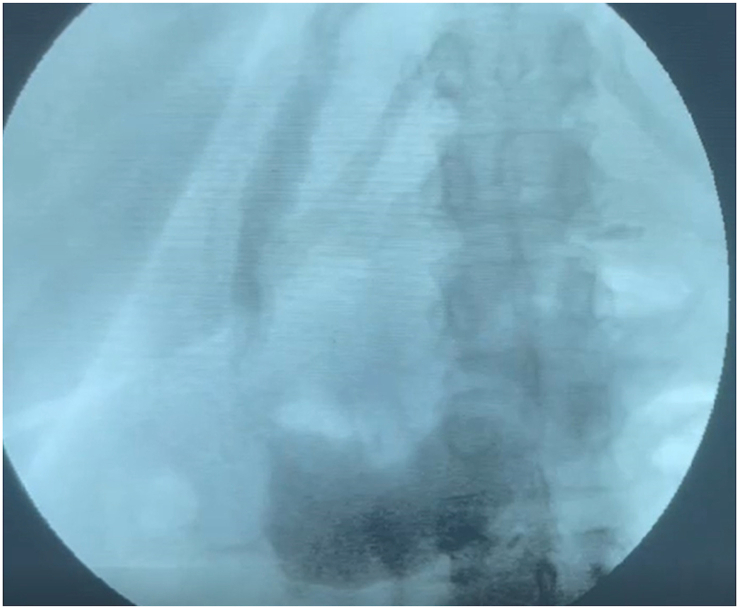
Gastrografin swallow, the gastric sleeve is seen on the left side of the abdomen.

## 3 Discussion

Obesity is a worldwide pandemic [1,2]. Over two-thirds of US adults aged 20 or older are overweight or obese, and regretfully, obesity is closely associated with an increased risk of comorbidities and overall mortality [2,3]. Medical treatment for obesity is generally ineffective regarding long-term weight control, and surgery has emerged as an essential strategy to ameliorate obesity-attributable comorbidities and as a powerful weight-loss tool [1,3]. This, combined a multidisciplinary team, will provide the patients all the necessary means to control their eating habits, comorbidities and promptly detect complications to perform a successful surgery with the sustained enhancement of quality of life and life expectancy [1,3]. Since obesity can affect almost everyone under the right conditions and due to the increasing number of obese patients predicted to elect bariatric surgery, individuals with rare anomalies such as situs inversus can be expected to seek weight loss surgery [4].

Ever since the first case reported in 1998, less than 40 cases of laparoscopic bariatric surgery on situs inversus patients have been published in the English literature [[5], [6], [7]]. Described for the first time by Fabricius et al., in 1600, situs inversus is an extremely rare pathology (1 every 5000 to 20.000 newborns) and is defined as the displacement of the intraabdominal organs 270° counterclockwise forming a mirror image [7]. Usually, both the thoracic and abdominal compartments are involved, but only one compartment can also be affected; most patients can live an ordinary life without complications, while others develop severe pulmonary, cardiovascular, or digestive abnormalities [[7], [8], [9]]. The most critical issue in this scenario is establishing the diagnosis in the preoperative period to plan for this unusual condition [3,10]. Diagnosis can be made through chest auscultation, X-rays, barium enema, ultrasound, endoscopy, or computed tomography [3,11]. If the diagnosis is made preoperative, it can help prevent pulmonary and cardiac complications and prepare for this rare surgery's mental and physical conditions and its equipment (trocar placement, positioning of the team, and overcoming the cognitive dissonance with this irregular anatomy), as operative times in these rare scenarios are greater by about 50% [1,2]. However, this condition does not seem to increase the risk of complications regarding the technical part, but if situs inversus is accompanied by Kartagener syndrome, the anesthesia team must be aware of possible difficulties [4,9,10].

Recognizing that Situs inversus is a rare congenital pathology in which the abdominal and thoracic organs are transposed from their usual position to the opposite side of the body. Since laparoscopic bariatric surgeries are highly demanding, variations from the normal anatomy (a mirror image) can challenge the medical team; therefore, preoperative diagnosis and highly trained surgeons will provide favorable outcomes to every patient.

## 4 Conclusions

By sharing this rare case and our experience managing this kind of patient, we believe that we can shine some light to guide future surgeons about how to handle similar patients as situs inversus does not change the management of obese patients. This case also highlights that the perioperative assessment of this and every patient is critical to ensure favorable results. As always, the experience and training of the surgical team will define the patient outcome because if there is a need to implement numerous techniques in order to provide adequate care, they must be performed by expert laparoscopic surgeons.

## Ethical approval

Written informed consent was obtained from the patient and ethical comitte for publication of this case report and accompanying images.

## Author contribution

GM and JZ analyzed and interpreted the patient data and did the surgical treatment. OP was a major contributor in writing the manuscript.MG prepared the figures. KR revised the manuscript and reviewed all the available data. All authors read and approved the final manuscript.

## Consent

Written informed consent was obtained from the patient for publication of this case report and accompanying images. A copy of the written consent is available for review by the Editor-in-Chief of this journal on request.

## Registration of research studies

NA.

## Guarantor

Gabriel A. Molina MD, Attending Surgeon at Hospital Ciba & Universidad San Francisco de Quito gabomolina32@gmail.com.

## Provenance and peer review

Not commissioned, externally peer-reviewed.

## Patient perspective

At first, the patient was scared about her treatment, whether it would hurt, and whether she could be “ok” nonetheless, since surgery was successful, she was grateful to the medical team.

## Sources of funding

None.

## Declaration of competing interest

We declare no conflict of interest.
